# Advances in Positron Emission Tomography Combined With Computed Tomography (PET/CT) Imaging in Cervical Cancer: From Metabolic Assessment to Fibroblast Activation Protein-Targeted Imaging

**DOI:** 10.7759/cureus.113158

**Published:** 2026-07-22

**Authors:** Sharjeel Usmani, Najeeb Ahmed, Ikram A Burney, Subhash Kheruka, Shah P Numani

**Affiliations:** 1 Radiology and Nuclear Medicine, Sultan Qaboos Comprehensive Cancer Care and Research Centre, Muscat, OMN; 2 Radiology, Molecular Imaging and Research Centre, Hull, GBR; 3 Oncology, Sultan Qaboos Comprehensive Cancer Care and Research Centre, Muscat, OMN; 4 Radiology, Augusta University, Augusta, USA

**Keywords:** ¹⁸f-fdg pet/ct, 68ga-fapi pet/ct, cancer-associated fibroblasts, cervical cancer, fibroblast activation protein

## Abstract

Positron emission tomography combined with computed tomography (PET/CT) has transformed the staging and management of cervical cancer, extending far beyond conventional anatomical imaging. ¹⁸F-fluorodeoxyglucose (¹⁸F-FDG) PET/CT currently serves as the standard-of-care functional imaging modality for initial staging, nodal mapping, treatment response assessment, and surveillance; however, its utility is constrained by limited spatial resolution for small nodal metastases, suboptimal specificity in inflammatory lesions, physiological urinary tracer excretion obscuring pelvic disease, and inability to characterize tumor microenvironmental composition. These limitations have driven the development of fibroblast activation protein inhibitor (FAPI) PET/CT, which capitalizes on the overexpression of fibroblast activation protein within the cancer-associated stroma of cervical tumors. FAPI-based imaging offers superior tumor-to-background contrast, improved delineation of primary lesions and metastatic deposits, and promising theranostic potential. This review traces the evolution from ¹⁸F-FDG metabolic imaging to FAPI-targeted PET/CT, reflecting a broader paradigm shift toward molecularly informed imaging in cervical cancer. Future directions include prospective clinical validation, radiomics integration, and the role of FAPI-PET in guiding adaptive radiotherapy and systemic treatment decisions.

## Introduction and background

Cervical cancer remains a significant global health burden, ranking as the fourth most common malignancy among women worldwide, with an estimated 660,000 new cases and over 350,000 deaths annually, disproportionately affecting low- and middle-income countries where screening programs remain limited [1,2]. Accurate staging and timely assessment of treatment response are critical determinants of survival, making imaging a cornerstone of clinical decision-making throughout the disease continuum.

Conventional imaging modalities, including computed tomography (CT) and magnetic resonance imaging (MRI), have long underpinned the staging and surgical planning of cervical cancer [3]. While MRI offers superior soft tissue resolution for local tumor assessment, and CT provides valuable anatomical detail for nodal and distant disease evaluation, both modalities are fundamentally limited by their reliance on morphological criteria [4]. Their inability to detect metabolically active but size-normal lymph nodes, differentiate post-treatment fibrosis from residual viable tumor, or characterize the biological activity of lesions represents a significant clinical gap [5].

¹⁸F-fluorodeoxyglucose (¹⁸F-FDG) positron emission tomography combined with computed tomography (PET/CT) has substantially advanced cervical cancer imaging by providing functional metabolic information alongside anatomical detail. Robust evidence supports its superiority over conventional imaging in lymph node staging, the detection of distant metastases, treatment response evaluation, and the surveillance for recurrence. Nevertheless, ¹⁸F-FDG PET/CT carries well-recognized limitations, including suboptimal specificity in inflammatory and infectious conditions, interference from physiological urinary excretion in the pelvis, and an inability to reflect tumor microenvironmental characteristics beyond glucose metabolism [6].

The recent emergence of fibroblast activation protein inhibitor (FAPI) PET/CT has opened a promising new frontier [7]. By targeting fibroblast activation protein (FAP) overexpressed on cancer-associated fibroblasts within the tumor stroma, FAPI tracers demonstrate high tumor-to-background contrast with low physiological pelvic uptake, offering complementary and potentially superior diagnostic performance [8,9]. This review aims to critically appraise the evolving evidence for PET/CT imaging in cervical cancer, highlighting the established role of ¹⁸F-FDG PET/CT, the emerging applications of FAPI-targeted PET/CT, and future directions for clinical integration. A narrative review methodology was adopted, with literature identified through searches of PubMed/MEDLINE, Scopus, and Google Scholar for publications available up to May 2026. The search strategy included combinations of keywords and Medical Subject Headings (MeSH) terms related to cervical cancer, PET/CT, ¹⁸F-FDG, FAPI, molecular imaging, staging, recurrence, prognosis, and theranostic applications. Articles were selected based on clinical relevance, methodological quality, and applicability to the scope of the review, with emphasis on guidelines, systematic reviews, meta-analyses, prospective studies, comparative studies, and larger retrospective cohorts. Additional relevant publications were identified through manual screening of reference lists from key studies.

## Review

Role of ¹⁸F-FDG PET/CT in cervical cancer

Cervical tumors exhibit upregulated glycolytic activity via the Warburg effect, driving preferential ¹⁸F-FDG accumulation proportional to metabolic tumor burden. ¹⁸F-FDG, a glucose analog, is taken up via GLUT transporters and phosphorylated, becoming metabolically trapped within tumor cells. This biological principle enables PET/CT to accurately detect primary lesions, identify nodal and distant metastases, assess treatment response, and surveil for recurrence, establishing it as the preferred functional imaging modality in cervical cancer management [10].

Clinical applications of ¹⁸F-FDG PET/CT in cervical cancer

Primary Tumor Evaluation

**Figure 1:**
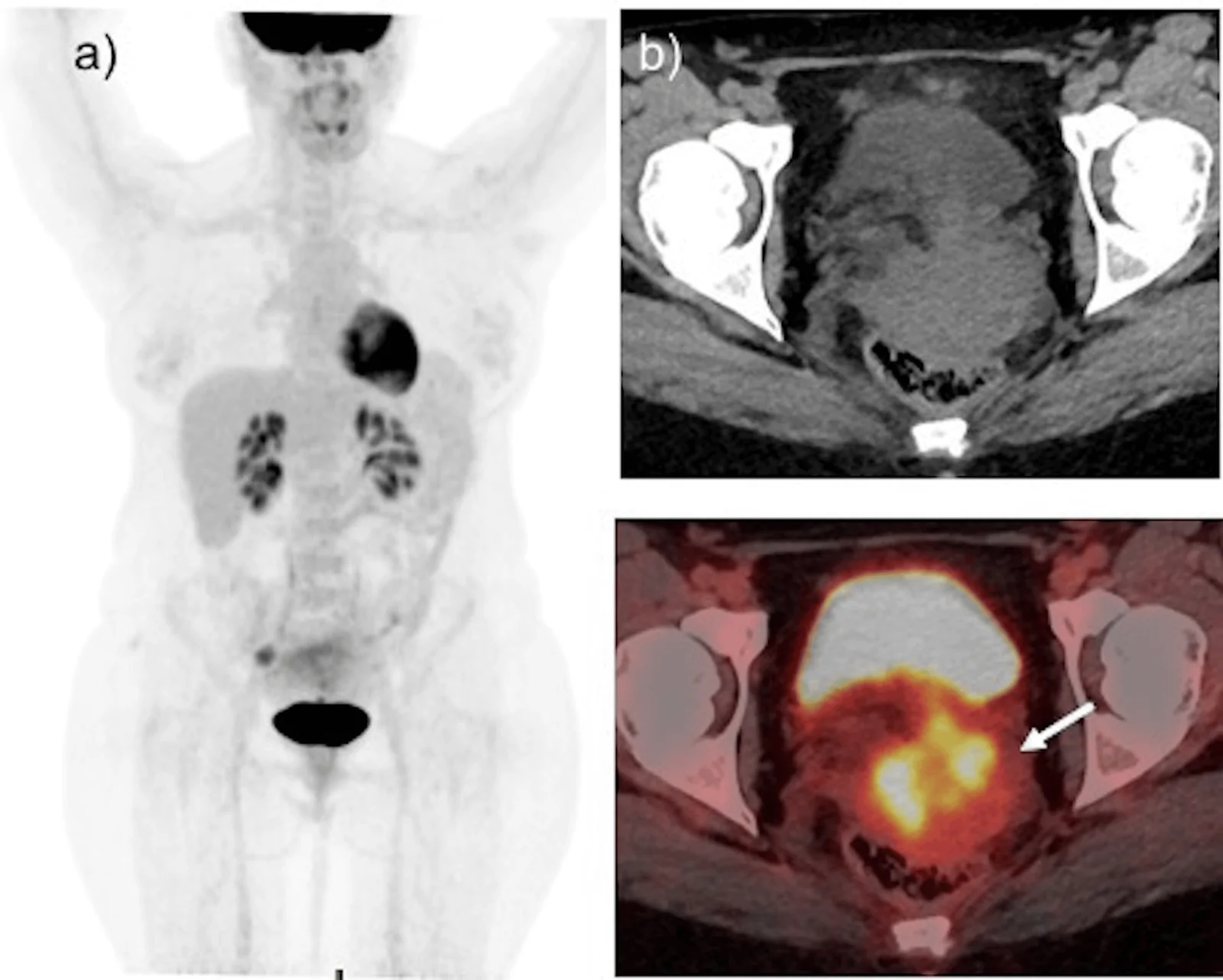
¹⁸F-FDG PET/CT in cervical cancer (a) MIP and (b) axial CT and fused ¹⁸F-FDG PET/CT images demonstrate an intensely ¹⁸F-FDG-avid primary cervical mass (white arrow). No metabolically active regional lymphadenopathy or distant metastatic disease is identified. ¹⁸F-FDG: ¹⁸F-fluorodeoxyglucose; PET: positron emission tomography; CT: computed tomography; MIP: maximum intensity projection

¹⁸F-FDG PET/CT characterizes the primary cervical tumor through metabolic activity quantification, with SUVmax correlating with tumor aggressiveness, histological grade, and prognosis. While MRI remains superior for assessing local parametrial and bladder invasion, PET/CT complements staging by identifying unsuspected synchronous lesions and establishing a metabolic baseline for subsequent treatment response evaluation. Studies have reported a sensitivity of 85-92% for primary lesion detection in stage Ib disease, with reliable identification of tumors measuring 2 cm or larger [11]. Furthermore, ¹⁸F-FDG PET/CT effectively delineates invasive tumor margins in cases of superior uterine cavity extension and inferior vaginal cuff involvement (Figure 1). However, its diagnostic performance is constrained by limited spatial resolution, partial volume effect, and susceptibility to false-negative findings in sub-centimeter lesions and micrometastatic deposits, representing an important limitation in early-stage and micrometastatic disease assessment [12].

Lymph Node Staging

Accurate nodal staging is a critical determinant of treatment strategy and survival in cervical cancer. ¹⁸F-FDG PET/CT demonstrates superior diagnostic performance over conventional CT and MRI by identifying metabolically active nodal metastases irrespective of size criteria, enabling more precise radiation field delineation, surgical nodal dissection planning, and individualized prognostic stratification [13]. In locally advanced cervical cancer (LACC), ¹⁸F-FDG PET/CT achieves pooled sensitivity and specificity of 72% and 96%, respectively, for the detection of pelvic and para-aortic lymph node metastases, reflecting its particular strength in confirming nodal involvement, while its moderate sensitivity underscores the persistent challenge of detecting small-volume and micrometastatic nodal disease [14].

Detection of Distant Metastases

¹⁸F-FDG PET/CT enables comprehensive whole-body oncological assessment in a single acquisition, facilitating the detection of distant metastases involving the lungs, liver, bones, and supraclavicular lymph nodes that would otherwise remain occult on conventional imaging. The identification of unsuspected distant disease carries profound clinical implications, shifting management intent from curative to palliative and avoiding futile radical interventions [15]. Reported diagnostic accuracy for distant metastasis detection demonstrates a specificity and negative predictive value of 97.7% and 93.1%, respectively, confirming its strength in excluding disseminated disease, while a moderate sensitivity of 54.8% and positive predictive value of 79.3% highlight the persisting challenge of detecting small-volume metastatic deposits [16].

Treatment Response Assessment

**Figure 2:**
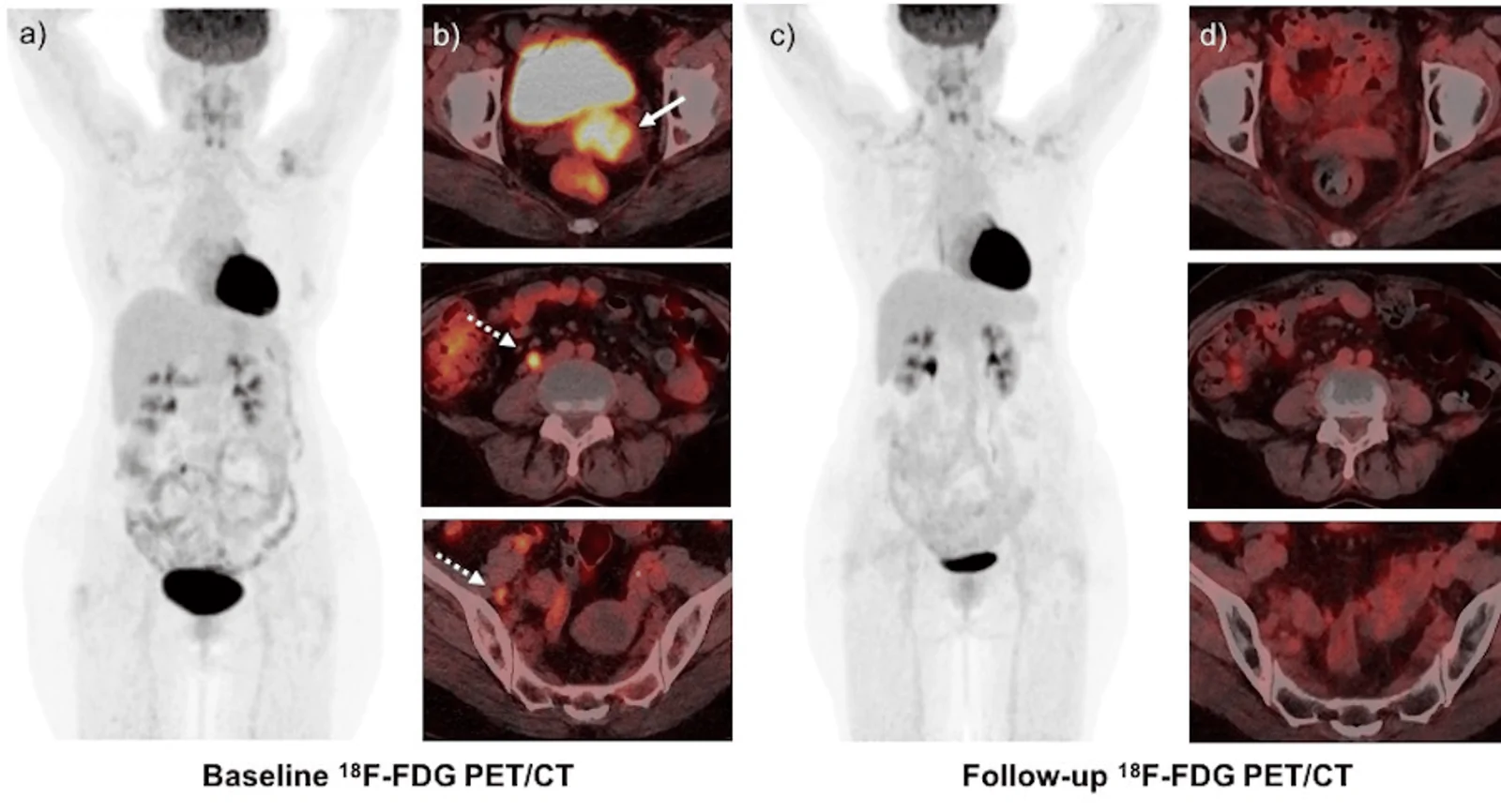
¹⁸F-FDG PET/CT in the treatment response assessment of cervical cancer Baseline (a) MIP and (b) fused ¹⁸F-FDG PET/CT images demonstrate an intensely ¹⁸F-FDG-avid primary cervical mass (solid arrow) with associated metastatic ¹⁸F-FDG-avid right common iliac and right external iliac lymph nodes (dotted arrow). Follow-up post-chemoradiation (c) MIP and (d) fused ¹⁸F-FDG PET/CT images show complete metabolic response, with the resolution of abnormal ¹⁸F-FDG uptake in both the primary cervical lesion and previously involved pelvic lymph nodes. ¹⁸F-FDG: ¹⁸F-fluorodeoxyglucose; PET: positron emission tomography; CT: computed tomography; MIP: maximum intensity projection

¹⁸F-FDG PET/CT reliably distinguishes metabolic complete response from residual viable tumor following chemoradiotherapy, providing prognostically significant information that surpasses the capabilities of anatomical imaging alone (Figure 2). Persistent or increasing ¹⁸F-FDG avidity post-treatment correlates with poor locoregional control and reduced overall survival, directly informing decisions regarding consolidation surgery, salvage radiotherapy, or systemic therapy escalation [17]. In accordance with the National Comprehensive Cancer Network (NCCN) guidelines, ¹⁸F-FDG PET/CT performed three months following the completion of chemoradiotherapy has become standard practice in locally advanced and metastatic cervical cancer. At this timepoint, the modality demonstrates diagnostic sensitivity and specificity of 97% and 99%, respectively, for the detection of residual tumor and metastatic disease [18], significantly outperforming conventional cross-sectional imaging and establishing PET/CT as the reference standard for post-treatment metabolic response evaluation.

Surveillance and Recurrence Detection

¹⁸F-FDG PET/CT is the modality of choice for detecting locoregional and distant recurrence in cervical cancer survivors presenting with rising tumor markers or clinical suspicion of relapse. Its capacity to differentiate metabolically active recurrent disease from post-treatment fibrosis, a distinction that remains challenging on conventional cross-sectional imaging, and to precisely localize sites of relapse for targeted salvage therapy confers significant diagnostic and therapeutic advantages in the post-treatment setting [19]. Notably, ¹⁸F-FDG PET/CT demonstrates substantially higher sensitivity than MRI for detecting suspected recurrent cervical cancer following radiotherapy, with reported values of 97.6% versus 80.1%, respectively [20], underscoring its superiority as the preferred surveillance imaging modality and supporting its routine incorporation into post-treatment follow-up protocols.

Radiotherapy Planning

¹⁸F-FDG PET/CT plays an increasingly integral role in radiotherapy planning for cervical cancer, enabling biologically guided target volume delineation that extends beyond the morphological boundaries of conventional anatomical imaging. By identifying metabolically active primary tumor volumes, involved pelvic and para-aortic nodes, and occult distant disease, PET/CT directly informs gross tumor volume (GTV) contouring and facilitates selective dose-escalation strategies to ¹⁸F-FDG-avid nodal deposits [21]. Clinically, ¹⁸F-FDG PET/CT has been shown to alter radiotherapy treatment plans in approximately 10-20% of patients through the detection of unsuspected extra-pelvic nodal metastases, directly expanding radiation fields and modifying treatment intent [22]. Furthermore, integration of PET-derived volumetric parameters, including metabolic tumor volume and total lesion glycolysis, into treatment planning workflows supports adaptive radiotherapy approaches, improving locoregional control while minimizing radiation dose to adjacent organs at risk.

Limitations of ¹⁸F-FDG PET/CT in cervical cancer

Despite its clinical utility, ¹⁸F-FDG PET/CT carries important limitations in cervical cancer imaging. False-positive findings frequently arise from benign inflammatory and infectious conditions, including post-radiation pelvic inflammation and reactive lymphadenopathy, reducing diagnostic specificity. Physiological urinary tracer excretion obscures adjacent pelvic structures, compromising the evaluation of the primary tumor and parametrial disease. Limited spatial resolution impairs reliable detection of sub-centimeter nodal metastases and micrometastatic deposits, representing a critical staging gap. Additionally, ¹⁸F-FDG provides no information on receptor expression or tumor microenvironmental composition, highlighting the need for molecularly targeted tracers to overcome these inherent biological and technical constraints.

Fibroblast activation imaging in cervical cancer

The emergence of FAPI PET/CT represents one of the most compelling recent advances in oncological imaging, exploiting the overexpression of FAP on cancer-associated fibroblasts within the tumor stroma to achieve highly specific tumor targeting with remarkably low physiological background activity. In cervical cancer, a malignancy characterized by a desmoplastic, fibroblast-rich tumor microenvironment, FAPI PET/CT holds particular promise as a superior alternative and complement to conventional ¹⁸F-FDG imaging.

Tumor microenvironment in cervical cancer

Cervical cancer is characterized by a dense desmoplastic stroma richly populated by cancer-associated fibroblasts, which actively promote tumor progression, angiogenesis, immune evasion, and resistance to chemoradiotherapy [23]. Unlike normal quiescent fibroblasts, cancer-associated fibroblasts selectively overexpress FAP, a transmembrane serine protease that remains largely absent in normal adult tissues but is abundantly expressed across the stromal compartment of cervical tumors. This selective FAP overexpression provides a compelling and highly specific molecular target for FAPI-based PET/CT imaging [24].

Mechanism of FAPI Tracers

FAPI tracers are small-molecule FAP-specific inhibitors radiolabeled with positron-emitting isotopes, most notably ⁶⁸Gallium (⁶⁸Ga-FAPI-04, ⁶⁸Ga-FAPI-46), that selectively bind with high affinity to FAP overexpressed on cancer-associated fibroblasts, enabling rapid tumor uptake and swift blood-pool clearance. This favorable pharmacokinetic profile translates into exceptionally high tumor-to-background contrast within minutes of administration, a distinct advantage over ¹⁸F-FDG, particularly in anatomical regions confounded by physiological tracer accumulation such as the pelvis.

Clinical applications of FAPI PET/CT in cervical cancer

Tumor Detection and Staging

⁶⁸Ga-FAPI PET/CT demonstrates avid and highly specific tracer accumulation within primary cervical tumors, reflecting dense cancer-associated fibroblast infiltration within the tumor stroma. Early evidence suggests superior primary lesion delineation compared to ¹⁸F-FDG, particularly in tumors with moderate glycolytic activity, enabling more precise tumor volume assessment and potentially refining FIGO staging accuracy in locally advanced disease [25]. In a proof-of-concept study, ⁶⁸Ga-FAPI-46 PET/CT demonstrated comparable or superior diagnostic performance to ¹⁸F-FDG PET/CT, successfully detecting all primary site lesions as well as distant metastatic deposits [26]. These preliminary findings highlight the potential of FAPI PET/CT to overcome the metabolic limitations of ¹⁸F-FDG, offering a more comprehensive and biologically specific staging tool in cervical cancer.

Improved Tumor-to-Background Contrast

A defining advantage of FAPI PET/CT over ¹⁸F-FDG in cervical cancer is its remarkably low physiological pelvic background activity, attributed to minimal FAP expression in normal uterine and bladder tissues. This translates into superior tumor-to-background contrast, overcoming the well-recognized limitation of urinary tracer excretion that frequently obscures primary cervical lesions and adjacent parametrial involvement on ¹⁸F-FDG PET/CT. ⁶⁸Ga-FAPI PET/CT has higher tracer accumulation in primary cervical cancer, with a mean SUV max of 15.22, which allows better delineation of the tumor [26].

Detection of Peritoneal and Nodal Disease

**Figure 3:**
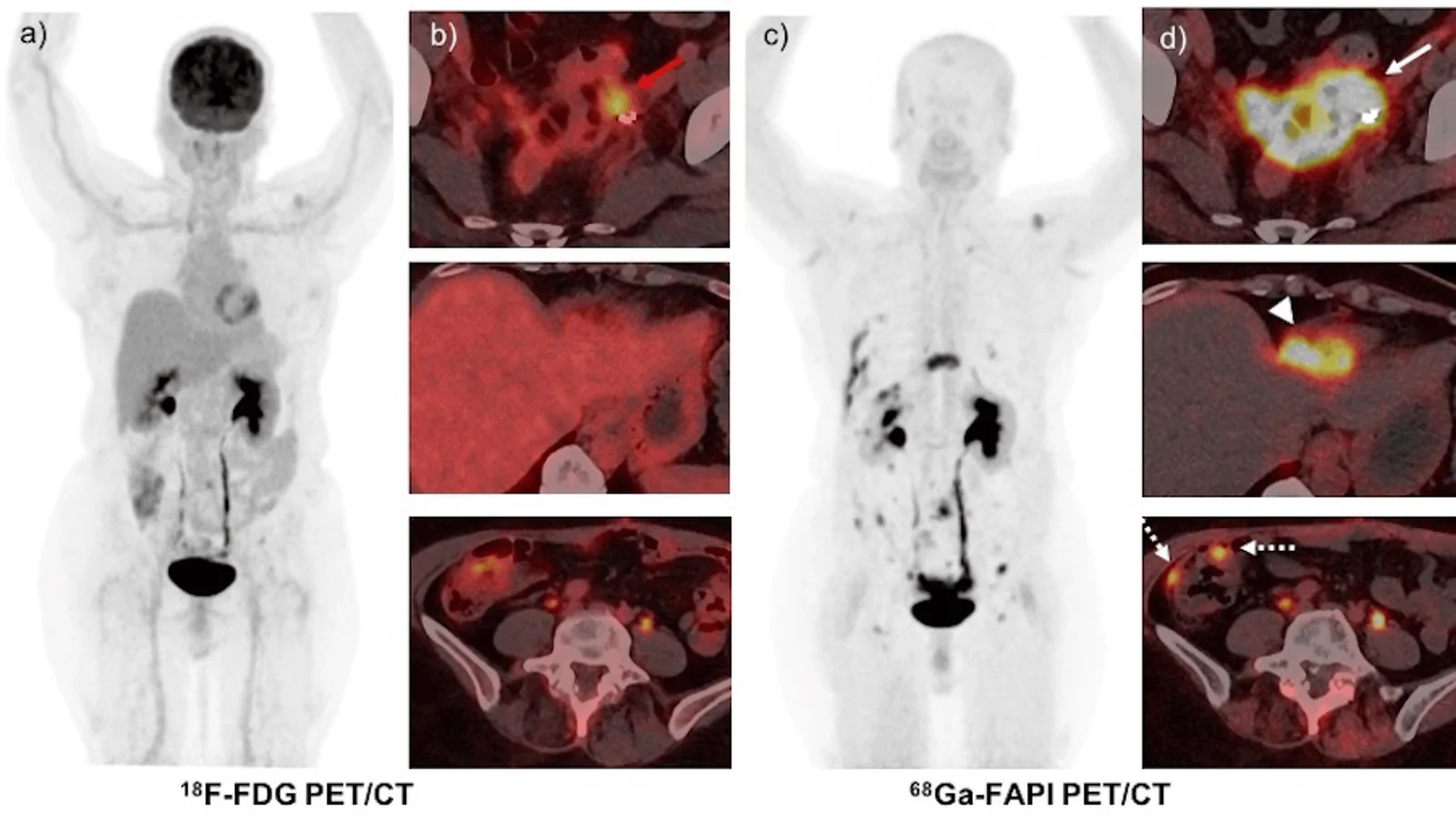
⁶⁸Ga-FAPI PET/CT in the evaluation of peritoneal disease (a) MIP and (b) fused ¹⁸F-FDG PET/CT images show a moderately avid infiltrative lesion (SUVmax 5.3, red arrow) at the vaginal stump abutting the rectum. (c) MIP and (d) fused ⁶⁸Ga-FAPI PET/CT images demonstrate a markedly avid lesion (SUVmax 15.1, white arrow) infiltrating the vaginal stump, rectosigmoid colon, and parametrial fat, with additional subdiaphragmatic, hepatic surface, bowel serosa (white dotted arrow), and diffuse peritoneal deposits. Findings highlight superior lesion detection and delineation with FAPI PET/CT, particularly for local recurrence and peritoneal carcinomatosis. ⁶⁸Ga: ⁶⁸Gallium; FAPI: fibroblast activation protein inhibitor; ¹⁸F-FDG: ¹⁸F-fluorodeoxyglucose; PET: positron emission tomography; CT: computed tomography; MIP: maximum intensity projection

⁶⁸Ga-FAPI PET/CT demonstrates promising sensitivity for detecting nodal metastases and peritoneal disease in cervical cancer, identifying FAP-avid deposits that may be missed by ¹⁸F-FDG due to small lesion size or insufficient glycolytic activity. In a small cohort of seven patients, Wegen et al. [27] reported accurate primary tumor delineation with ⁶⁸Ga-FAPI-46 PET/CT, demonstrating significantly higher tumor-to-background ratios compared to ¹⁸F-FDG PET/CT in both primary tumors and lymph node metastases, with the detection of a greater number of lesions overall. Furthermore, ⁶⁸Ga-FAPI PET/CT has demonstrated superior sensitivity over ¹⁸F-FDG for the evaluation of peritoneal carcinomatosis, attributable to its low physiological background activity, high tracer avidity for FAP-expressing stromal cells, and consistently elevated tumor-to-background ratio [28,29]. These findings carry significant clinical implications for treatment stratification, extended radiation field planning, and identification of patients who may benefit from systemic therapeutic escalation (Figure 3).

Comparison of ¹⁸F-FDG and FAPI PET/CT in cervical cancer

Emerging comparative studies suggest FAPI PET/CT achieves superior diagnostic performance over ¹⁸F-FDG, particularly for low-¹⁸F-FDG-avid lesions where glycolytic activity is insufficient for reliable detection. FAPI tracers identify stromal-rich deposits missed by ¹⁸F-FDG, demonstrating higher tumor-to-background ratios, fewer false-positive inflammatory findings, and improved diagnostic confidence in both primary staging and recurrence detection [30]. In a head-to-head comparison of ¹⁸F-FDG and ⁶⁸Ga-FAPI-04 PET/CT in 35 cervical cancer patients, Shu et al. [31] reported comparable tracer uptake patterns for primary tumor and lymph node detection; however, a key advantage of ⁶⁸Ga-FAPI-04 emerged in its superior ability to differentiate metastatic from reactive lymph nodes, a clinically critical distinction that directly influences treatment planning. Corroborating these findings, a recent prospective study by Lyu et al. [32] in early-stage cervical cancer patients (T stage ≤2a2) demonstrated equivalent primary tumor detection between ⁶⁸Ga-FAPI-04 PET/MR and ¹⁸F-FDG PET/CT, while nodal metastasis detection was significantly superior with FAPI, achieving a sensitivity of 100% compared to 59.1% with ¹⁸F-FDG (p=0.004). Collectively, these findings underscore the particular diagnostic advantage of FAPI PET/CT in nodal staging, where accurate disease characterization carries the greatest impact on treatment stratification and outcomes (Table 1).

**Table 1:** Comparative analysis of ¹⁸F-FDG and FAPI PET/CT modalities in cervical cancer ¹⁸F-FDG: ¹⁸F-fluorodeoxyglucose; PET: positron emission tomography; CT: computed tomography; FAPI: fibroblast activation protein inhibitor; FAP: fibroblast activation protein

Parameter	¹⁸F-FDG PET/CT (metabolic imaging)	FAPI PET/CT (fibroblast activation imaging)
Biological target	Glucose metabolism (glycolysis)	Cancer-associated fibroblasts (FAP expression in tumor stroma)
Mechanism	Increased ¹⁸F-FDG uptake via GLUT transporters and hexokinase trapping	Binding to fibroblast activation protein in tumor microenvironment
Tumor-to-background contrast	Moderate	Excellent
Strengths	Widely available; standardized; strong evidence base; guideline-recommended. High sensitivity (especially for nodal and distant metastases)	Superior lesion contrast; detects lesions with low ¹⁸F-FDG avidity; highlights tumor stroma. Very high (excellent tumor-to-background ratio)
Weaknesses	False positives; limited specificity; reduced sensitivity in small/low-grade lesions	Limited availability; early clinical data; lack of standardized protocols
Suggested clinical scenarios	Initial staging; treatment response; surveillance; detection of metastases	Cases with equivocal ¹⁸F-FDG findings; low-¹⁸F-FDG-avid tumors; need for high-contrast imaging

Current evidence and ongoing research

Early clinical studies evaluating FAPI PET/CT in cervical cancer have demonstrated encouraging diagnostic performance; however, it is acknowledged that available data remain limited, with few prospective studies specifically designed for this malignancy. Preliminary findings consistently suggest superior nodal metastasis detection with ⁶⁸Ga-FAPI compared to ¹⁸F-FDG PET/CT, indicating its potential as a complementary staging tool and possible alternative to surgical nodal staging particularly in high-risk patients who may be spared an invasive procedure. Beyond diagnostics, FAP-targeted theranostics pairing ⁶⁸Ga-labeled imaging with therapeutic radionuclide-conjugated FAPI agents represents an emerging and potentially personalized treatment avenue in FAP-overexpressing refractory cervical malignancies, though clinical evidence in this specific context remains at an early stage [33]. We have endeavored to address this evidence gap transparently throughout the manuscript, and we emphasize the need for larger, prospective, multicenter studies to further establish the diagnostic accuracy, clinical utility, and theranostic potential of FAPI PET/CT across all stages of cervical cancer management before definitive recommendations can be made.

Future directions

The future of PET imaging in cervical cancer lies in multimodal innovation and personalized strategies. Hybrid tracers combining metabolic and FAP-targeted properties may overcome the complementary limitations of individual agents, while dual-tracer imaging protocols promise comprehensive tumor characterization in a single setting. PET/MRI integration offers simultaneous functional and superior soft tissue anatomical assessment, particularly valuable for local disease evaluation and radiotherapy/brachytherapy planning. Radiomics and artificial intelligence applied to PET datasets hold significant potential for automated lesion segmentation, prognostic modeling, and treatment response prediction, enabling data-driven personalization of care. Theranostic platforms pairing diagnostic FAPI imaging with targeted radionuclide therapy represent a transformative precision oncology frontier, warranting urgent prospective investigation in cervical cancer to establish efficacy and optimal patient selection criteria.

## Conclusions

PET/CT imaging has become an important component of cervical cancer management, with ¹⁸F-FDG PET/CT remaining the established clinical standard for staging, treatment response assessment, and surveillance. However, limitations related to specificity, physiological pelvic background activity, and limited characterization of the tumor microenvironment have stimulated interest in emerging fibroblast-targeted imaging approaches such as FAPI PET/CT. Early studies suggest that FAPI PET/CT may provide complementary information, particularly in selected clinical scenarios where ¹⁸F-FDG-based imaging has limitations, and may offer future opportunities for theranostic applications. Nevertheless, current evidence in cervical cancer remains limited, and the clinical impact of FAPI PET/CT requires further validation. Larger prospective, multicenter studies are needed to establish its diagnostic accuracy, clinical utility, prognostic value, and potential role in personalized theranostic strategies before routine integration into clinical practice and guidelines.
